# Out-of-Hospital Cardiac Arrest in Southern Italy: A Retrospective Analysis of 11,653 Cases

**DOI:** 10.3390/jcdd13030146

**Published:** 2026-03-23

**Authors:** Luca Gregorio Giaccari, Pasquale Sansone, Nicola D’Angelo, Daniele Antonaci, Eva Epifani, Luciana Mascia, Maria Caterina Pace, Vincenzo Pota, Gaetano Tammaro

**Affiliations:** 1 Service of Anesthesia and Intensive Care, Vito Fazzi Hospital, 73100 Lecce, Italy; 2 Department of Woman, Child, General and Specialized Surgery, University of Campania “Luigi Vanvitelli”, 80138 Naples, Italy; 3 Emergency Medical Service 118, Local Health Authority (ASL) Lecce, 73100 Lecce, Italy; 4 Service of Hospital Management, “Veris Delli Ponti” Hospital, 73020 Scorrano, Italy; 5 Department of Experimental Medicine, University of Salento, 73100 Lecce, Italy

**Keywords:** out-of-hospital cardiac arrest, ventricular fibrillation, asystole, circadian rhythm, COVID-19, return of spontaneous circulation, emergency medical services

## Abstract

(1) Background: Out-of-hospital cardiac arrest (OHCA) remains a major public health issue, with survival largely determined by the initial rhythm and timeliness of resuscitation. Comprehensive population-based data are essential for guiding prevention, emergency medical services (EMS) planning, and improving outcomes. (2) Methods: We performed a retrospective observational study of all adult OHCA cases managed by EMS in Lecce (Italy) between January 2013 and March 2025. Demographics, arrest circumstances, initial rhythm, time intervals, and return of spontaneous circulation (ROSC) were analyzed across age, sex, temporal, and pandemic-related strata. Rhythm classification followed European Resuscitation Council guidelines. (3) Results: A total of 11,653 cases were analyzed (mean age 76.8 ± 15.5 years, 56.6% male). Asystole (AS) was the predominant rhythm (88.7%), followed by ventricular fibrillation (VF, 7.6%), pulseless electrical activity (PEA, 1.3%), and pulseless ventricular tachycardia (pVT, 0.08%). VF was more common in younger and male patients, while AS increased with age. Hour-level analysis revealed circadian peaks: VF in late afternoon and AS in early morning. Pandemic analysis showed reduced VF and increased AS during COVID-19, with partial recovery post-pandemic. ROSC occurred in 3.47% overall, strongly associated with shockable rhythms. EMS response times were stable across day–night and pandemic phases. (4) Conclusions: AS dominates OHCA presentations, especially among the elderly, whereas VF remains the strongest predictor of ROSC. Circadian variation at the hourly level suggests potential for EMS optimization. Pandemic-related shifts in rhythm highlight the vulnerability of the chain of survival to societal disruptions. Strengthening bystander CPR, expanding AED availability, and tailoring EMS strategies remain key priorities for improving OHCA outcomes.

## 1. Introduction

Out-of-hospital cardiac arrest (OHCA) remains a major public health challenge, with an annual incidence of 55–110 cases per 100,000 inhabitants in Europe [1]. Despite advances in pre-hospital care, survival rates remain low, largely due to the high prevalence of non-shockable rhythms and the time-sensitive nature of resuscitation [2,3]. The initial rhythm recorded by emergency medical services (EMS) is a strong predictor of outcome: shockable rhythms such as ventricular fibrillation (VF) and pulseless ventricular tachycardia (pVT) are associated with higher survival compared with asystole (AS) or pulseless electrical activity (PEA) [4].

The distribution of presenting rhythms is influenced by patient demographics, comorbidities, arrest circumstances, and EMS response intervals [5,6]. Age and sex differences are well documented: younger and male patients more often present with VF/pVT, whereas AS and PEA are more frequent in older individuals [7]. Temporal patterns have also been observed, with circadian and seasonal variations possibly reflecting autonomic tone, cardiovascular triggers, and environmental stressors [8,9]. Characterizing these epidemiological features is essential for prevention strategies, EMS planning, and improved outcomes.

The COVID-19 pandemic added new challenges, with reports of increased OHCA incidence, longer EMS response times, reduced bystander cardiopulmonary resuscitation (CPR), and shifts toward non-shockable rhythms during the first outbreak [10,11]. Evidence on post-pandemic rhythm distribution and outcomes, however, remains limited.

In this study, we report data from a large cohort of OHCA and analyze key clinical, demographic, and temporal aspects to identify potential associations and targets for improving OHCA management.

## 2. Materials and Methods

### 2.1. Study Design and Setting

We conducted a retrospective observational study including all OHCA cases attended by the Servizio di Emergenza Urgenza 118 within the Azienda Sanitaria Locale (ASL) di Lecce, Apulia region, Southern Italy, between 1 January 2013 and 16 March 2025. The retrospective observational study complied with the principles of the Declaration of Helsinki, and ethical approval was obtained from the local committee of the “Vito Fazzi” Hospital (protocol no. 14/2018, approval date 15 January 2018). According to the institutional policies, no additional IRB approval was required for the extension of the retrospective data collection period until 16 March 2025, as the study design remained unchanged and the approval covered ongoing retrospective data analysis of eligible patients within the institution.

### 2.2. Inclusion and Exclusion Criteria

All OHCA cases recorded in the EMS electronic database during the study period were considered eligible. We excluded patients younger than 18 years, traumatic arrests, and cases lacking essential data such as age. Only the first rhythm documented by EMS personnel upon arrival at the scene was used for rhythm classification.

### 2.3. Data Collection

Data were retrieved from the standardized “Scheda Intervento” forms routinely completed by EMS teams. Variables included patient demographics, date and time of arrest, initial presenting rhythm, number of shocks delivered, occurrence and timing of ROSC, and operational time intervals: emergency call reception (T0), dispatch (T1), arrival on scene (T2), and time to ROSC. For subgroup analyses, age was categorized into four predefined groups (18–39, 40–59, 60–79, and ≥80 years) to reflect clinically relevant life stages and to facilitate comparison with previous OHCA epidemiological studies. Rhythm classification followed European Resuscitation Council (ERC) guidelines [12]. Initial rhythms were classified into four categories: ventricular fibrillation (VF), pulseless ventricular tachycardia (pVT), pulseless electrical activity (PEA), and asystole (AS), according to European Resuscitation Council definitions. For descriptive purposes, rhythms were also grouped into shockable (VF and pVT) and non-shockable (PEA and AS) categories. In cases where the EMS documentation did not allow reliable classification of the initial rhythm, the rhythm was categorized as “unspecified”. ROSC was defined as the restoration of spontaneous circulation documented by EMS personnel during resuscitation efforts, regardless of duration, as recorded in the EMS intervention report.

For temporal analyses, the 24-h day was divided into daytime (08:00–19:59) and nighttime (20:00–07:59) periods. In addition to this dichotomous comparison, circadian variation was explored by analyzing the distribution of initial rhythms across individual hours of the day (00:00–23:59). Events were also categorized according to weekday versus weekend occurrence. Seasonal variation was assessed by grouping cases into four meteorological seasons: spring (March–May), summer (June–August), autumn (September–November), and winter (December–February). To assess the impact of the COVID-19 pandemic, three distinct phases were defined: pre-pandemic (until 29 February 2020), intra-pandemic (1 March 2020–31 December 2021), and post-pandemic (from 1 January 2022 onwards).

In this EMS-based registry, post-resuscitation hospital outcomes (e.g., survival to hospital admission, survival to discharge, or neurological status) were not systematically available. Therefore, ROSC documented by EMS personnel was used as the primary outcome measure.

### 2.4. Statistical Analysis

Continuous variables such as age, time intervals, and number of shocks were summarized as mean ± standard deviation and median with interquartile range, while categorical variables were reported as counts and percentages. Normality was checked, but given the skewed distribution of intervals, non-parametric tests were mainly applied. Rhythm distributions across demographic, temporal, and pandemic strata were compared using Pearson’s χ^2^ test, with Fisher’s exact test applied when assumptions were violated. Continuous variables were compared with Mann–Whitney U tests for two groups and Kruskal–Wallis tests for multiple groups, while normally distributed data were assessed with ANOVA. ROSC times were analyzed both as continuous and categorical variables (≤20, 21–40, >40 min), with trend tests applied when appropriate. Circadian variation was evaluated using contingency tables comparing rhythm categories across hourly intervals, and standardized residuals were examined to identify specific cells contributing to significant deviations from expected frequencies. Missing data were handled through a combination of case exclusion and explicit categorization. For variables considered essential for the primary analyses, cases with missing values were excluded from the respective analyses. For other variables, missing values were retained and categorized as “unspecified” in order to preserve sample size and maintain transparency in reporting. Cases with missing age were excluded from the dataset, as age was considered an essential variable for demographic stratification. For other variables, including sex and initial rhythm, records were retained in the dataset and categorized as “unspecified” when information was unavailable or ambiguous in the EMS documentation. Analyses of rhythm distribution were therefore performed using available rhythm classifications, while cases with unspecified rhythm were excluded from rhythm-specific subgroup comparisons but retained in overall cohort counts. For time-to-ROSC analyses, only cases with complete time information were included. Statistical analyses were performed using R (R Foundation for Statistical Computing, Vienna, Austria) and IBM SPSS Statistics version 27 (IBM Corp., Armonk, NY, USA). A two-sided *p* value < 0.05 was considered statistically significant.

## 3. Results

A total of 11,653 adult patients experienced OHCA. Of these, 6595 were male (56.6%) and 5028 female (43.1%), with sex unspecified in 30 cases. The mean age of the population was 76.8 ± 15.5 years (range 18–107), with a median of 81 years, reflecting a predominantly elderly cohort. The baseline characteristics of the OHCA cohort are summarized in Table 1. 894 (7.7%) exhibited a shockable rhythm, while 10,498 (90.1%) presented with a non-shockable rhythm. In 271 cases (2.2%), rhythm could not be classified due to missing or ambiguous data. These cases were retained in the overall cohort counts but excluded from rhythm-specific subgroup analyses to avoid distortion of percentage estimates. These cases were retained in the overall cohort counts and are reported as “unspecified rhythm” in Table 1. Among patients with shockable rhythms, 888 received shocks, with a mean of 3.6 ± 3.2 shocks (range 1–20). The most common presenting rhythm was AS, occurring in 10,334 patients (88.68%). VF was second with 885 cases (7.59%), PEA occurred in 154 patients (1.32%), and pVT in 9 cases (0.08%). In 271 cases (2.33%), the presenting rhythm was unspecified. AS patients (*n* = 10,334) had a mean age of 77.5 ± 15.3 years; 5708 were male (55.2%) and 4601 female (44.5%). VF cases (*n* = 885) had a mean age of 68.6 ± 15.6 years, with a male predominance (72.7% vs. 27.0%). PEA cases (*n* = 154) had a mean age of 79.3 ± 12.9 years, with 92 males (59.7%) and 61 females (39.6%). pVT was rare (*n* = 9), with a mean age of 75.3 ± 15.3 years, affecting 3 males (33.3%) and 6 females (66.7%).

**Figure 1:**
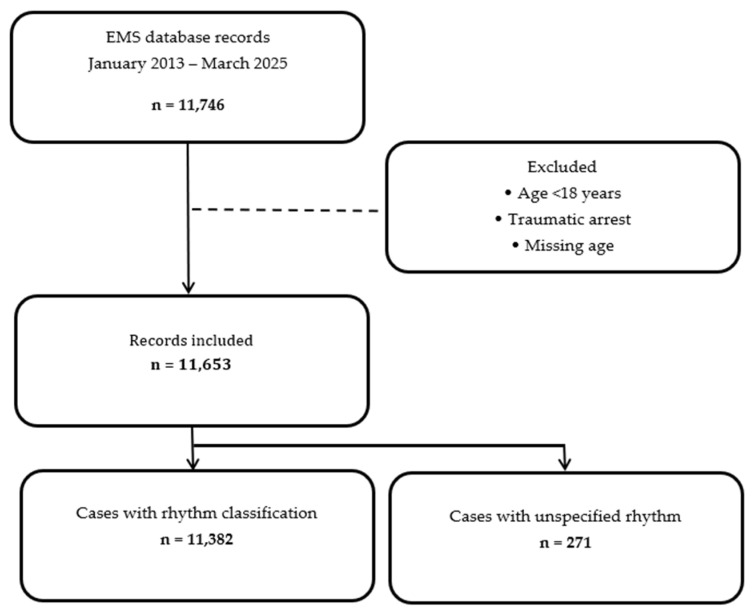
Study flow diagram of case selection.

The case selection process and dataset structure are summarized in Figure 1.

Age. In the 18–39-year group (*n* = 374), AS occurred in 326 cases (87.2%), VF in 37 (9.9%), and PEA in 4 (1.1%); no pVT was recorded. Among patients aged 40–59 years (*n* = 1263), AS accounted for 1008 cases (79.8%), VF for 206 (16.3%), PEA for 7 (0.6%), and pVT for 2 (0.2%). In the 60–79-year group (*n* = 3631), AS occurred in 3127 cases (86.1%), VF in 377 (10.4%), PEA in 48 (1.3%), and pVT in 2 (0.1%). In patients aged ≥80 years (*n* = 6376), AS accounted for 5866 cases (92.0%), VF for 263 (4.1%), PEA for 95 (1.5%), and pVT for 5 (0.1%). Rhythm distribution varied significantly by age (χ^2^ = 186.66, df = 12, *p* < 0.0001).

Sex. Among females, AS occurred in 4601 cases (93.8%), VF in 239 (4.9%), PEA in 61 (1.2%), and pVT in 6 (0.1%). Among males, AS occurred in 5708 cases (88.6%), VF in 643 (10.0%), PEA in 92 (1.4%), and pVT in 3 (0.05%). Rhythm distribution differed significantly between sexes (χ^2^ = 104.50, df = 3, *p* < 0.0001).

Time of day. During daytime (*n* = 7682), AS was observed in 6975 cases (90.8%), VF in 587 (7.6%), PEA in 111 (1.4%), and pVT in 9 (0.1%). At night (*n* = 3700), AS occurred in 3359 cases (90.8%), VF in 298 (8.1%), PEA in 43 (1.2%), and no pVT. Differences between daytime and nighttime were not significant (χ^2^ = 6.36, df = 3, *p* = 0.095). A small number of cases had incomplete timestamp information and were therefore not included in the day–night comparison.

**Figure 2:**
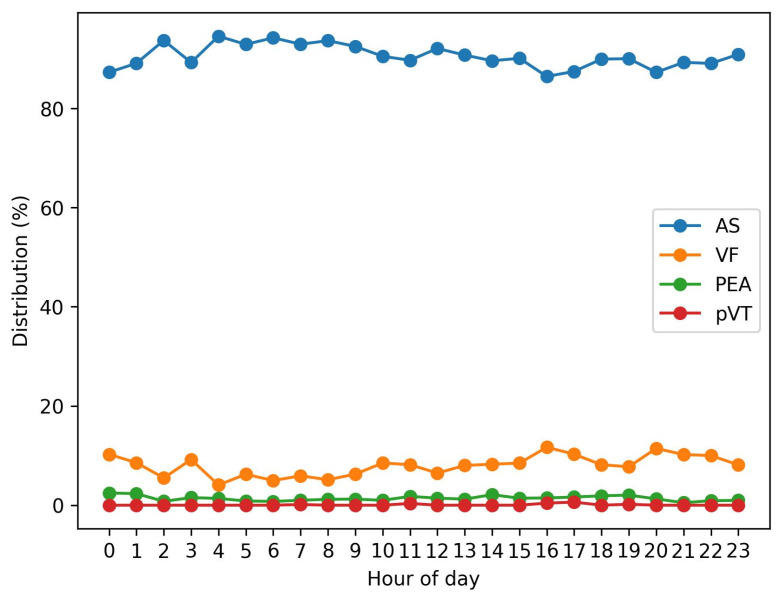
Hourly distribution of initial cardiac rhythms.

Hour-by-hour analysis showed significant variation (χ^2^ = 120.01, df = 69, *p* = 0.00014). The hourly distribution of rhythms is shown in Figure 2. VF peaked at 16:00 (11.7%), 20:00 (11.4%), and 17:00 (10.3%). AS remained high throughout the day but relatively lower in late afternoon (86.5% at 16:00) compared with early morning (>94% at 02:00–06:00). PEA showed modest peaks at 14:00 (2.1%), 19:00 (2.0%), and 00:00 (2.5%). pVT appeared sporadically at 07:00, 11:00, 16:00, 17:00, and 19:00.

Weekdays vs. weekends. On weekdays (*n* = 7981), AS occurred in 7228 cases (90.6%), VF in 631 (7.9%), PEA in 116 (1.5%), and pVT in 6 (0.1%). On weekends (*n* = 3378), AS accounted for 3087 cases (91.4%), VF for 251 (7.4%), PEA for 37 (1.1%), and pVT for 3 (0.1%). No significant differences were observed (χ^2^ = 3.18, df = 3, *p* = 0.364).

Season. In autumn (*n* = 2471), AS occurred in 2228 cases (90.2%), VF in 209 (8.5%), PEA in 34 (1.4%), and no pVT. In spring (*n* = 2778), AS occurred in 2514 (90.5%), VF in 224 (8.1%), PEA in 38 (1.4%), and pVT in 2 (0.1%). In summer (*n* = 2734), AS occurred in 2489 (91.0%), VF in 206 (7.5%), PEA in 34 (1.2%), and pVT in 5 (0.2%). In winter (*n* = 3376), AS occurred in 3084 (91.4%), VF in 243 (7.2%), PEA in 47 (1.4%), and pVT in 2 (0.1%). Seasonal differences were not significant (χ^2^ = 9.85, df = 9, *p* = 0.363).

COVID-19 periods. In the pre-COVID phase (*n* = 6033), AS occurred in 5399 cases (89.5%), VF in 516 (8.6%), PEA in 114 (1.9%), and pVT in 4 (0.1%). During the COVID phase (*n* = 1887), AS occurred in 1746 (92.5%), VF in 127 (6.7%), PEA in 12 (0.6%), and pVT in 2 (0.1%). In the post-COVID phase (*n* = 3439), AS occurred in 3170 (92.2%), VF in 239 (7.0%), PEA in 27 (0.8%), and pVT in 3 (0.1%). Differences were significant (χ^2^ = 41.50, df = 6, *p* < 0.001), with higher VF and fewer AS cases post-COVID.

Dispatch and arrival times. The dispatch interval (call to dispatch) was 3.6 ± 2.2 min (median 3.0, IQR 2–4; range 0–14) during daytime and 3.76 ± 2.36 min (median 3.0, IQR 2–5; range 0–13) at night. The difference was not significant (Mann–Whitney U = 3942.0, *p* = 0.431).

The arrival interval (call to arrival) was 11.6 ± 5.2 min (median 11.0, IQR 8–14; range 0–35) during daytime and 11.7 ± 5.7 min (median 11.0, IQR 8–15; range 0–32) at night. The difference was not significant (Mann–Whitney U = 3929.0, *p* = 0.452).

ROSC. A total of 404 patients (3.47%) achieved ROSC. The characteristics of ROSC cases are summarized in Table 2. The mean age was 67.6 ± 15.7 years, median 69 years. Of these, 276 were male (68.3%) and 128 female (31.7%).

By initial rhythm, 223 patients (55.2%) presented with VF, 4 (1.0%) with pVT, 106 (26.2%) with AS, and 19 (4.7%) with PEA; in 52 cases (12.9%), rhythm was unspecified. For shockable rhythms, 792 shocks were delivered, with a mean of 3.5 ± 3.1 per case.

In 393 valid cases, mean time from arrival to ROSC was 30.3 ± 16.6 min (range 2–96). Eleven ROSC cases lacked complete time-stamp data in the EMS records and were therefore excluded from the time-interval analysis. By rhythm, ROSC times were: VF 30.3 ± 18.2 min (range 2–96, *n* = 223), pVT 25.5 ± 8.7 (16–37, *n* = 4), AS 31.1 ± 14.7 (range 2–80, *n* = 106), and PEA 31.3 ± 13.6 (range 14–65, *n* = 19). One-way ANOVA showed no significant differences (*p* = 0.922).

Age. Among the 404 patients achieving ROSC, 276 were male (68.3%) and 128 female (31.7%). When stratified by initial rhythm, VF accounted for the majority of ROSC cases in both sexes. Among VF ROSC cases (*n* = 223), 175 (78.5%) occurred in males and 46 (20.6%) in females. For asystole-related ROSC (*n* = 106), 56 cases (52.8%) occurred in males and 47 (44.3%) in females. Rhythm distribution differed significantly between sexes (χ^2^ test, *p* < 0.0001).

Mean ROSC times increased with age: 25.5 ± 10.6 min (range 11–50) in 18–39 years, 27.3 ± 16.0 (range 2–81) in 40–59 years, 31.3 ± 17.7 (range 5–93) in 60–79 years, and 32.3 ± 15.8 (range 2–96) in ≥80 years. The trend was not statistically significant (ANOVA, *p* = 0.081).

When categorized, ROSC times differed significantly (χ^2^ = *p* = 0.0076). In 18–39 years, 36.8% achieved ROSC ≤ 20 min, 57.9% in 21–40 min, and 5.3% in >40 min. In 40–59 years, the proportions were 42.1%, 37.9%, and 20.0%. In 60–79 years, 29.5%, 47.4%, and 23.1%. In ≥80 years, 17.9%, 54.7%, and 27.4%.

Sex. Among females (*n* = 128), AS occurred in 47 cases (45.6%), VF in 46 (20.8%), PEA in 6 (33.3%), and pVT in 3 (75.0%). Among males (*n* = 276), AS accounted for 56 (54.4%), VF for 175 (79.2%), PEA for 12 (66.7%), and pVT for 1 (25.0%). Rhythm distribution differed significantly (χ^2^, *p* < 0.0001).

Mean ROSC time was longer in females (32.7 ± 15.9 min, range 2–91) than males (29.2 ± 16.8, range 2–96), with statistical significance (*t*-test, *p* = 0.046). Categorized times also differed (χ^2^, *p* = 0.00143): in females, 17.6% achieved ROSC ≤ 20 min, 56.8% within 21–40 min, and 25.6% in >40 min; in males, 35.4%, 43.2%, and 21.2%.

Time of day. VF predominated during both day (59.7%, *n* = 160) and night (69.0%, *n* = 60). AS accounted for 30.2% (*n* = 81) during day and 24.1% (*n* = 21) at night. PEA occurred in 5.2% (*n* = 14) and 4.6% (*n* = 4), respectively, while pVT was only observed during daytime (1.5%, *n* = 4). No significant association was found (χ^2^, *p* = 0.379).

Mean ROSC times were 30.0 ± 16.6 min (range 2–93, *n* = 297) by day and 31.3 ± 16.6 (range 8–96, *n* = 96) by night, with no significant difference (*t*-test, *p* = 0.515). Categorical analysis showed 31.0%, 46.1%, and 22.9% versus 26.0%, 52.1%, and 21.9% for ≤20, 21–40, and >40 min (χ^2^, *p* = 0.558).

Weekdays vs. weekends. On weekdays, VF was most common (65.5%, *n* = 165), followed by AS (25.0%, *n* = 63), PEA (5.6%, *n* = 14), and pVT (1.2%, *n* = 3). On weekends, VF occurred in 54.0% (*n* = 54), AS in 39.0% (*n* = 39), PEA in 4.0% (*n* = 4), and pVT in 1.0% (*n* = 1). Differences were not significant (χ^2^, *p* = 0.082).

Mean ROSC times were 30.0 ± 16.6 min (range 2–96, *n* = 278) on weekdays and 30.6 ± 15.9 (range 5–81, *n* = 113) on weekends (*t*-test, *p* = 0.770). Categorical proportions were similar (28.8%, 50.0%, 21.2% vs. 31.9%, 42.5%, 25.6%; χ^2^, *p* = 0.385).

Season. VF predominated in all seasons, ranging from 57.5% in winter (*n* = 46) to 68.5% in autumn (*n* = 61). AS varied between 25.2% and 32.6%, highest in winter (*n* = 28). PEA was uncommon (1.1–9.0%), and pVT was rare, observed only in summer (*n* = 3) and winter (*n* = 1). Seasonal distribution showed no significant association (χ^2^, *p* = 0.236).

Mean ROSC times were 31.0 ± 17.9 min in winter (*n* = 93), 29.7 ± 16.2 in spring (*n* = 95), 29.7 ± 15.2 in summer (*n* = 103), and 30.4 ± 16.4 in autumn (*n* = 100). ANOVA showed no significant difference (*p* = 0.936). Categorical analysis also showed no variation (χ^2^, *p* = 0.895).

COVID-19 periods. VF proportions increased across periods: 57.5% pre-COVID (*n* = 131), 63.9% intra-COVID (*n* = 23), and 74.7% post-COVID (*n* = 65). AS declined from 33.3% pre-COVID (*n* = 76) to 22.2% intra-COVID (*n* = 8) and 20.7% post-COVID (*n* = 18). PEA occurred in 6.6% pre-COVID (*n* = 15) and 3.4% post-COVID (*n* = 3), but not intra-COVID. pVT was rare, with 2 intra-COVID cases. Differences were significant (χ^2^, *p* = 0.0067).

Mean ROSC times were 29.5 ± 15.6 min pre-COVID (*n* = 259), 31.8 ± 14.6 intra-COVID (*n* = 38), and 31.5 ± 18.8 post-COVID (*n* = 94), with no significant difference (ANOVA, *p* = 0.495).

## 4. Discussion

This population-based study, comprising 11,653 adult out-of-hospital cardiac arrest (OHCA) cases, provides a detailed overview of initial rhythm distribution, temporal patterns, and resuscitation outcomes across demographic, temporal, and pandemic-related strata. The findings highlight significant variations associated with age, sex, and circadian patterns, as well as a notable shift in rhythm profile during the COVID-19 period.

AS emerged as the most frequent initial rhythm (88.7%), followed by VF (7.6%), PEA (1.3%), and pVT (0.08%). The number of pVT cases was limited; therefore, findings related to this rhythm should be interpreted with caution. For clinical interpretation, VF and pVT are commonly grouped as shockable rhythms, as both are treated with defibrillation and share similar prognostic implications. However, we report the two rhythms separately to preserve the original EMS documentation and to provide a complete description of rhythm distribution in this population. These results align with large-scale European registry data [13,14], in which non-shockable rhythms account for the majority of OHCA presentations, particularly in older populations. The mean patient age in our study (76.8 years) and the predominance of cases in the ≥80-year group (54.7%) likely explain the low proportion of VF, given the known association between age, unwitnessed arrests, and non-shockable presentations [15]. VF occurrence was more common in younger age groups and in males, peaking at 16.3% in patients aged 40–59 years and 10% in men overall. This pattern mirrors established epidemiological trends linking VF to coronary artery disease and acute myocardial infarction [15,16]. Conversely, AS prevalence increased progressively with age, exceeding 92% in patients ≥ 80 years, suggesting that degenerative conduction disease, comorbidity burden, and delayed EMS activation play a larger role in the elderly [17]. The relatively low ROSC rate observed in our cohort (3.47%) should be interpreted in the context of the specific characteristics of the study population and EMS registry structure. In our dataset, all adult OHCA cases attended by EMS were included regardless of resuscitation attempt or presumed etiology. Consequently, the cohort contains a large proportion of very elderly patients, with a mean age of 76.8 years and more than half of cases occurring in individuals aged ≥80 years. Advanced age is strongly associated with unwitnessed arrests, delayed recognition, and a higher prevalence of non-shockable rhythms, all of which reduce the likelihood of ROSC. In addition, some international OHCA registries restrict analysis to resuscitation-attempted cases or presumed cardiac etiologies, which typically increases the proportion of shockable rhythms and survival outcomes. The broader inclusion criteria used in the present study likely contribute to both the high prevalence of asystole and the lower overall ROSC rate observed in this population.

The hourly analysis revealed significant non-uniformity in VF, AS, and PEA occurrence over the 24-hour cycle. VF showed distinct peaks in the late afternoon (16:00 and 20:00), whereas AS proportions were highest in the early morning (02:00–06:00). These findings are in line with previous studies reporting circadian variation in OHCA incidence and arrhythmia onset, attributed to fluctuations in sympathetic tone, cortisol secretion, platelet aggregability, and triggering of ischemic events [18,19]. Importantly, no significant day–night difference emerged when dichotomizing the data, suggesting that coarse temporal groupings may obscure clinically relevant peaks. Hour-level granularity could therefore be valuable for EMS planning, particularly if resource allocation is adapted to anticipated high-incidence periods [20,21].

The absence of significant variation in VF, AS, and PEA between weekdays and weekends, or across seasons, contrasts with some studies reporting increased cardiac arrest incidence during winter months, potentially linked to cold-induced cardiovascular stress [22,23]. One explanation could be the urban context of our population, where indoor climate control mitigates seasonal effects, and the predominance of elderly patients whose activity levels are less seasonally variable.

A notable finding was the shift in VF and AS distribution during and after the COVID-19 pandemic. The intra-pandemic period was marked by a reduction in VF proportion and an increase in AS, consistent with early reports of reduced bystander CPR, longer EMS response times, and increased incidence of unwitnessed arrests [24,25]. Conversely, the post-pandemic phase showed an increased VF proportion and reduced AS prevalence, suggesting partial restoration of pre-pandemic resuscitation dynamics and public engagement [26,27].

Given the large sample size of the cohort, statistically significant differences may occur even when the absolute differences between groups are relatively small. Therefore, the results should be interpreted primarily in terms of their clinical relevance and epidemiological meaning rather than statistical significance alone.

ROSC was achieved in 3.47% of all OHCA cases, with VF representing 55.2% of these successes. The overall ROSC rate observed in our cohort appears lower than that reported in several contemporary OHCA registries. This difference should be interpreted in light of important methodological and population-related factors. First, our study included all EMS-attended OHCA cases, without restriction to presumed cardiac etiology and without limiting the analysis to cases in which resuscitation was actively attempted. In contrast, many large registries report outcomes only for resuscitation-attempted cases or for arrests of presumed cardiac origin, which typically results in higher ROSC rates. Additionally, the population included in our study was characterized by advanced age and a high burden of comorbidities, factors that are known to negatively influence resuscitation outcomes. These differences in case selection and population characteristics likely contribute to the comparatively lower ROSC rate observed in our cohort.

The strong association between shockable rhythms (VF, pVT) and survival aligns with prior evidence [28], underscoring the prognostic weight of initial presentation. The age gradient in ROSC characteristics—shorter times in younger patients, longer times in older adults—may be explained by greater physiological reserve, fewer comorbidities, and higher likelihood of witnessed arrest in younger cohorts [29]. Sex differences were also apparent: males had a higher proportion of early ROSC (≤20 min), while females more often achieved ROSC in the 21–40 min range. While the reasons for this difference are unclear, possible explanations include differences in cardiovascular pathophysiology, hormonal influences, and variations in EMS intervention timing or intensity [30].

Dispatch and arrival times were consistent between day and night, suggesting robust EMS capacity across shifts. This uniformity likely contributed to the lack of significant differences in ROSC rates and durations between diurnal periods. However, the low overall ROSC rate underscores the ongoing challenge of OHCA management, particularly in elderly populations with predominantly AS presentations [31].

These findings have several implications. First, targeted prevention strategies should consider demographic risk profiles: younger males may benefit most from coronary artery disease prevention and rapid defibrillation access, while elderly populations require strategies to reduce delays in recognition and EMS activation [32]. Second, the detection of circadian peaks in VF suggests that EMS resource optimization—such as strategic AED placement and targeted public messaging—could be focused during late afternoon hours [33]. Third, the pandemic-related VF and AS shifts highlight the vulnerability of the chain of survival to societal disruption and reinforce the importance of maintaining bystander CPR capacity even during public health crises [34].

Further work is needed to explore biological mechanisms underlying circadian variation in VF and AS occurrence, investigate the influence of comorbidities and medication profiles on rhythm type, evaluate whether hour-level EMS deployment adjustments could improve outcomes, and monitor post-pandemic trends to determine if VF and AS distributions stabilize or continue to evolve [35,36].

This study has several limitations that should be acknowledged. First, the retrospective design relies on routinely collected EMS records, which may contain incomplete, inconsistent, or ambiguously documented information. Data were extracted from operational EMS reports that were not originally designed for research purposes, and therefore some variables were occasionally missing or incompletely recorded. Although age was required for inclusion in the analysis, a small number of records lacked complete information for other variables such as sex or initial rhythm. In these cases, the variables were classified as “unspecified” and retained in the dataset to avoid unnecessary data loss. Similarly, certain time-related variables were unavailable in a limited number of ROSC cases, which reduced the sample size for analyses involving time intervals. Second, the EMS database used for this study does not systematically capture several key Utstein variables that are known to strongly influence OHCA outcomes. In particular, information on witnessed status, bystander cardiopulmonary resuscitation (CPR), arrest location, first monitored rhythm before EMS arrival, and presumed etiology was not consistently available. The absence of these variables limits the ability to adjust for important confounders and restricts the interpretation of the observed associations between demographic or temporal factors and rhythm distribution or ROSC. Third, the study population is characterized by a relatively advanced age, with a large proportion of cases occurring in patients aged ≥80 years. This demographic structure likely contributes to the high prevalence of non-shockable rhythms observed in the cohort and to the relatively low overall ROSC rate compared with some contemporary OHCA registries that include younger populations or only resuscitation-attempted cases. Consequently, caution is required when comparing the present findings with those from other regional or international registries. Fourth, given the exploratory nature of several subgroup analyses, the possibility of Type I error due to multiple statistical comparisons should be considered, particularly when interpreting findings related to less frequent rhythm categories. Fifth, because the study was conducted within a single EMS system in Southern Italy, the findings may not be fully generalizable to other geographic settings with different demographic characteristics, EMS organization, or resuscitation practices. Finally, ROSC was the only outcome measure available in the EMS registry. Information on survival to hospital admission, survival to discharge, and neurological outcome was not available. Consequently, the results describe early resuscitation success rather than long-term clinical outcomes.

Despite these limitations, the large sample size and long observation period provide valuable epidemiological insights into rhythm distribution and temporal patterns of OHCA within a real-world EMS population.

## 5. Conclusions

In this elderly cohort, asystole was the most frequent rhythm, while ventricular fibrillation remained the main predictor of early ROSC. Hourly analysis revealed clear circadian variation, not captured by simple day–night comparisons. Rhythm shifts during the COVID-19 pandemic highlighted the vulnerability of the chain of survival. Overall, these findings reinforce the prognostic weight of the initial rhythm, the importance of early defibrillation for shockable rhythms, and the need for tailored strategies for elderly patients with non-shockable arrests. From a public health perspective, strengthening bystander CPR capacity, expanding AED accessibility, and adapting EMS deployment to circadian rhythm patterns represent key priorities for improving outcomes after OHCA.

## Figures and Tables

**Table 1:** Characteristics of OHCA.

Variable	*n* (%)	AS *n* (%)	VF *n* (%)	PEA *n* (%)	pVT *n* (%)
Total	11,653 *	10,334 (88.7)	885 (7.6)	154 (1.3)	9 (0.1)
Age (years)					
18–39	374	326 (87.2)	37 (9.9)	4 (1.1)	0
40–59	1263	1008 (79.8)	206 (16.3)	7 (0.6)	2 (0.2)
60–79	3631	3127 (86.1)	377 (10.4)	48 (1.3)	2 (0.1)
≥80	6376	5866 (92.0)	263 (4.1)	95 (1.5)	5 (0.1)
Sex					
Male	6446	5708 (88.6)	643 (10.0)	92 (1.4)	3 (0.05)
Female	4907	4601 (93.8)	239 (4.9)	61 (1.2)	6 (0.1)
Time of day					
Day	7682	6975 (90.8)	587 (7.6)	111 (1.4)	9 (0.1)
Night	3700	3359 (90.8)	298 (8.1)	43 (1.2)	0
Weekday/Weekend					
Weekdays	7981	7228 (90.6)	631 (7.9)	116 (1.5)	6 (0.1)
Weekends	3378	3087 (91.4)	251 (7.4)	37 (1.1)	3 (0.1)
Season					
Spring	2778	2514 (90.5)	224 (8.1)	38 (1.4)	2 (0.1)
Summer	2734	2489 (91.0)	206 (7.5)	34 (1.2)	5 (0.2)
Autumn	2471	2228 (90.2)	209 (8.5)	34 (1.4)	0
Winter	3376	3084 (91.4)	243 (7.2)	47 (1.4)	2 (0.1)
COVID-19 period					
Pre-COVID	6033	5399 (89.5)	516 (8.6)	114 (1.9)	4 (0.1)
Intra-COVID	1887	1746 (92.5)	127 (6.7)	12 (0.6)	2 (0.1)
Post-COVID	3439	3170 (92.2)	239 (7.0)	27 (0.8)	3 (0.1)

* An amount of 271 (2.3%) cases had missing or ambiguous rhythm documentation in the EMS database and were therefore classified as unspecified rhythm. Percentages for rhythm categories are calculated based on available rhythm classifications, while totals include all OHCA cases in the cohort.

**Table 2:** Characteristics of ROSC.

Variable	*n* (%)	VF *n* (%)	AS *n* (%)	PEA *n* (%)	pVT *n* (%)
Total	404 (100) *	223 (55.2)	106 (26.2)	19 (4.7)	4 (1.0)
Age group					
18–39	19 (4.7)	12 (63.2)	5 (26.3)	0	0
40–59	95 (23.5)	65 (81.3)	13 (16.3)	0	1 (1.3)
60–79	173 (42.8)	104 (58.1)	47 (26.3)	9 (5.0)	1 (0.6)
≥80	106 (26.2)	40 (42.6)	38 (40.4)	9 (9.6)	2 (2.1)
Sex					
Male	276 (68.3)	175 (79.2)	56 (54.4)	12 (66.7)	1 (25.0)
Female	128 (31.7)	46 (20.8)	47 (45.6)	6 (33.3)	3 (75.0)
Time of day					
Day	297 (73.5)	160 (59.7)	81 (30.2)	14 (5.2)	4 (1.5)
Night	96 (23.8)	60 (69.0)	21 (24.1)	4 (4.6)	0
Weekday/Weekend					
Weekdays	278 (68.8)	165 (65.5)	63 (25.0)	14 (5.6)	3 (1.2)
Weekends	113 (28.0)	54 (54.0)	39 (39.0)	4 (4.0)	1 (1.0)
Season					
Spring	95 (23.5)	63 (66.3)	26 (27.4)	4 (4.2)	0
Summer	103 (25.5)	66 (64.1)	28 (27.2)	6 (5.8)	3 (2.9)
Autumn	100 (24.8)	61 (68.5)	28 (28.3)	2 (2.0)	0
Winter	93 (23.0)	46 (57.5)	28 (30.1)	7 (7.5)	1 (1.1)
COVID-19 period					
Pre-COVID	259 (64.1)	131 (57.5)	76 (33.3)	15 (6.6)	0
Intra-COVID	38 (9.4)	23 (63.9)	8 (22.2)	0	2 (5.3)
Post-COVID	94 (23.3)	65 (74.7)	18 (20.7)	3 (3.4)	0

* Rhythm classification was unavailable in 52 ROSC cases due to incomplete documentation in the EMS records. These cases were included in overall ROSC counts but excluded from rhythm-specific subgroup analyses.

## Data Availability

The datasets generated and analyzed during the current study are available from the corresponding author on reasonable request.

## Abbreviations

The following abbreviations are used in this manuscript:

ANOVA	Analysis of Variance
AS	Asystole
ASL	Azienda Sanitaria Locale
AED	Automated External Defibrillator
CPR	Cardiopulmonary Resuscitation
EMS	Emergency Medical Services
ERC	European Resuscitation Council
IQR	Interquartile Range
OHCA	Out-of-Hospital Cardiac Arrest
pVT	Pulseless Ventricular Tachycardia
PEA	Pulseless Electrical Activity
ROSC	Return of Spontaneous Circulation
VF	Ventricular Fibrillation

## References

[1] Gräsner J.T., Wnent J., Herlitz J., Perkins G.D., Lefering R., Tjelmeland I., Koster R.W., Masterson S., Rossell-Ortiz F., Maurer H. (2020). Survival after out-of-hospital cardiac arrest in Europe—Results of the EuReCa TWO study. Resuscitation.

[2] Berdowski J., Berg R.A., Tijssen J.G., Koster R.W. (2010). Global incidences of out-of-hospital cardiac arrest and survival rates: Systematic review of 67 prospective studies. Resuscitation.

[3] Sasson C., Rogers M.A., Dahl J., Kellermann A.L. (2010). Predictors of survival from out-of-hospital cardiac arrest: A systematic review and meta-analysis. Circ. Cardiovasc. Qual. Outcomes.

[4] Kudenchuk P.J., Brown S.P., Daya M., Rea T., Nichol G., Morrison L.J., Leroux B., Vaillancourt C., Wittwer L., Callaway C.W. (2016). Amiodarone, lidocaine, or placebo in out-of-hospital cardiac arrest. N. Engl. J. Med..

[5] Daya M.R., Schmicker R.H., Zive D.M., Rea T.D., Nichol G., Buick J.E., Brooks S., Christenson J., MacPhee R., Craig A. (2015). Out-of-hospital cardiac arrest survival improving over time: Results from the Resuscitation Outcomes Consortium (ROC). Resuscitation.

[6] Hollenberg J., Herlitz J., Lindqvist J., Riva G., Bohm K., Rosenqvist M., Svensson L. (2008). Improved survival after out-of-hospital cardiac arrest is associated with an increase in proportion of emergency crew–witnessed cases and bystander CPR. Circulation.

[7] Strömsöe A., Svensson L., Axelsson Å.B., Claesson A., Göransson K.E., Nordberg P., Herlitz J. (2015). Improved outcome in Sweden after out-of-hospital cardiac arrest and possible association with improvements in every link in the chain of survival. Eur. Heart J..

[8] Willich S.N., Levy D., Rocco M.B., Tofler G.H., Stone P.H., Muller J.E. (1987). Circadian variation in the incidence of sudden cardiac death in the Framingham Heart Study population. Am. J. Cardiol..

[9] Arntz H.R., Willich S.N., Schreiber C., Brüggemann T., Stern R., Schultheiß H.-P. (2000). Diurnal, weekly and seasonal variation of sudden death. Eur. Heart J..

[10] Baldi E., Sechi G.M., Mare C., Canevari F., Brancaglione A., Primi R., Klersy C., Palo A., Contri E., Ronchi V. (2020). COVID-19 kills at home: The close relationship between the epidemic and the increase of out-of-hospital cardiac arrests. Eur. Heart J..

[11] Scquizzato T., Landoni G., Paoli A., Lembo R., Fominskiy E., Kuzovlev A., Likhvantsev V., Zangrillo A. (2020). Effects of COVID-19 pandemic on out-of-hospital cardiac arrests: A systematic review. Resuscitation.

[12] Soar J., Böttiger B.W., Carli P., Couper K., Deakin C.D., Djärv T., Lott C., Olasveengen T., Paal P., Pellis T. (2021). European Resuscitation Council Guidelines 2021: Adult Advanced Life Support. Resuscitation.

[13] Gräsner J.T., Lefering R., Koster R.W., Masterson S., Böttiger B.W., Herlitz J., Wnent J., Tjelmeland I.B.M., Ortiz F.R., Maurer H. (2016). EuReCa ONE—27 nations, ONE Europe, ONE registry: A prospective one-month analysis of out-of-hospital cardiac arrest outcomes in 27 countries in Europe. Resuscitation.

[14] Gräsner J.T., Wnent J., Herlitz J., Perkins G.D., Lefering R., Tjelmeland I.B., Beinf B., Böttigerh B.W., Rosell-Ortizi F., Nolan J.P. (2021). Epidemiology of cardiac arrest in Europe: Results from the EuReCa TWO study. Resuscitation.

[15] Thakur R.K., Hoffmann R.G., Olson D.W., Joshi R., Tresch D.D., Aufderheide T.P., Ip J.H. (1996). Circadian variation in sudden cardiac death: Effects of age, sex, and initial cardiac rhythm. Ann. Emerg Med..

[16] Pecková M., Fahrenbruch C.E., Cobb L.A., Hallstrom A.P. (1998). Circadian variations in the occurrence of cardiac arrests. Circulation.

[17] van Dongen L.H., de Goede P., Moeller S., Eroglu T.E., Folke F., Gislason G., Blom M.T., Elders P.J.M., Torp-Pedersen C., Tan H.L. (2021). Temporal variation in out-of-hospital cardiac arrest occurrence in individuals with or without diabetes. Resusc. Plus.

[18] Savopoulos C., Ziakas A., Hatzitolios A., Delivoria C., Kounanis A., Mylonas S., Tsougas M., Psaroulis D. (2006). Circadian rhythm in sudden cardiac death: A retrospective study of 2665 cases. Angiology.

[19] Delisle B.P. (2021). Understanding circadian mechanisms of sudden cardiac death. Circ. Arrhythm. Electrophysiol..

[20] Yao P.C., Li M.H., Chen M., Che Q.J., Fei Y.D., Li G.L., Sun J., Wang Q.S., Wu Y.B., Yang M. (2024). Circadian variation pattern of sudden cardiac arrest in a Chinese community. Open Heart.

[21] Ho A.F.W., Hao Y., Pek P.P., Shahidah N., Yap S., Ng Y.Y., Wong K.D., Lee E.J., Khruekarnchana P., Wah W. (2019). Outcomes and modifiable resuscitative characteristics amongst pan-Asian out-of-hospital cardiac arrest occurring at night. Medicine.

[22] Škrlec I., Milic J., Heffer M. (2021). Sex differences in circadian clock genes and myocardial infarction. J. Cardiovasc. Dev. Dis..

[23] Mayuga K.A., Thattassery E., Taneja T., Karha J., Subacius H., Goldberger J., Kadish A. (2010). Circadian and Gender Effects on Repolarization in Healthy Adults: A Study Using Harmonic Regression Analysis. Ann. Noninvasive Electrocardiol..

[24] Delisle B.P. (2025). Circadian influences on sudden cardiac death and arrhythmias. J. Mol. Cell. Cardiol..

[25] Fabianek J., Felzen M., Riester K.R., Beckers S.K., Rossaint R., Schröder H., Pitsch M. (2024). The impact of smartphone-dispatched CPR-trained volunteers on OHCA outcomes is influenced by patient age. Sci. Rep..

[26] Majewski D., Ball S., Bailey P., Bray J., Finn J. (2022). Trends in out-of-hospital cardiac arrest incidence, patient characteristics and survival over 18 years in Perth, Western Australia. Resusc. Plus.

[27] Lee N., Jung S., Ro Y.S., Park J.H., Hwang S.-S. (2024). Spatiotemporal analysis of out-of-hospital cardiac arrest in Korea. J. Korean Med. Sci..

[28] Goniewicz M., Misztal-Okońska P., Hertelendy A.J., Goniewicz K., Burkle F.M. (2024). EMS response times influence ROSC: A study from Poland. Acta Pol. Emerg. Med..

[29] Ahn J.Y., Ryoo H.W., Moon S., Jung H., Park J., Lee W.K., Kim J.-Y., Lee D.E., Kim J.H., Lee S.-H. (2023). Prehospital factors associated with out-of-hospital cardiac arrest outcomes in a metropolitan city: A 4-year multicenter study. BMC Emerg. Med..

[30] Kornfehl A., Krammel M., Grassmann D., de Zordo M., Brock R., Veigl C., Adler R., Dunkl S., Gatterbauer M., Gonzo P. (2025). The impact of additional special emergency medical service units on non-traumatic adult out-of-hospital cardiac arrest outcomes in a high-resource metropolitan area. Resusc. Plus.

[31] Morin F., Trottier H., Stiell I.G., Vaillancourt C. (2022). Deployment of “super lay-rescuers” with AEDs to improve OHCA survival. Resuscitation.

[32] Starks M.A., Chu J., Leung K.B., Blewer A.L., Simmons D., Hansen C.M., Joiner A., Cabañas J.G., Harmody M.R., Nelson R.D. (2024). Combinations of First Responder and Drone Delivery to Achieve 5-Minute AED Deployment in OHCA. JACC Adv..

[33] Kelters I.R., Koop Y., Young M.E., Daiber A., van Laake L.W. (2025). Circadian rhythms in cardiovascular disease. Eur. Heart J..

[34] Ghazal B.Z., Kaur A., Sandhu G.S., Sharma S. (2023). Biological sex, circadian rhythms, and cardiovascular risk: A narrative review. Cardiol. Rev..

[35] Müller J.E., Stone P.H., Turi Z.G., Rutherford J.D., Czeisler C.A., Parker C., Poole W.K., Passamani E., Roberts R., Robertson T. (1985). Circadian variation in the frequency of onset of acute myocardial infarction. N. Engl. J. Med..

[36] Brooks S.C., Schmicker R.H., Rea T.D., Aufderheide T.P., Davis D.P., Morrison L.J., Sahni R., Sears G.K., Griffiths D.E., Sopko G. (2010). Out-of-hospital cardiac arrest frequency and survival: Evidence for circadian variability in OHCA frequency. Resuscitation.

